# The effect of fentanyl combined with linezolid on in-hospital mortality in mechanically ventilated patients: A retrospective cohort study

**DOI:** 10.1371/journal.pone.0337648

**Published:** 2026-01-28

**Authors:** Jia-Hui Niu, Xiao-Jiao Cui, Min Chen, Jin-Qi Li, Xiao-Qing Yi

**Affiliations:** 1 Department of Pharmacy, Personalized Drug Research and Therapy Key Laboratory of Sichuan Province, Sichuan Provincial People’s Hospital, University of Electronic Science and Technology of China,; 2 Sichuan Academy of Medical Sciences, Sichuan Provincial People’s Hospital, School of Medicine, University of Electronic Science and Technology of China, Chengdu, China, No. 32, West Second Section, First Ring Road, Chengdu, China; Children's National Hospital, George Washington University, UNITED STATES OF AMERICA

## Abstract

**Background:**

The combination of linezolid and opioid drugs such as fentanyl may increase the risk of serotonin syndrome, but its impact on in-hospital mortality is not yet clear. The aim of this study is to investigate the effect of simultaneous use of fentanyl and linezolid on in-hospital mortality in mechanically ventilated patients.

**Method:**

Based on the MIMIC-IV database, 3339 patients receiving mechanical ventilation were enrolled and divided into three groups: the group receiving linezolid simultaneously (n = 43), the group receiving linezolid within 14 days (n = 22) and the group that did not use linezolid (n = 3274). Use multivariate Cox regression analysis to analyze in-hospital mortality rates and adjust for confounding factors.

**Result:**

The in-hospital mortality rate of the group receiving linezolid simultaneously was 37.2% (16/43), the group receiving linezolid within 14 days was 40.9% (9/22), and the group that did not use linezolid was 22.9% (751/3274). The in-hospital mortality rate of the group receiving linezolid simultaneously was significantly higher than that of the group that did not use linezolid (hazard ratio [HR], 1.56; 95% CI, 1 ~ 2.43; P = 0.049). There was no statistically significant difference in in-hospital mortality rate among the group receiving linezolid within 14 days and the group that did not use linezolid (hazard ratio [HR], 1.01; 95% CI, 0.5 ~ 2.05; P = 0.968). Subgroup analysis showed that there was no interaction between different groups at baseline (age, gender, race, BMI, liver disease, and kidney disease) (interaction p-value > 0.05).

**Conclusion:**

In this post-hoc analysis, we found an association between the combined use of fentanyl and linezolid and increased in-hospital mortality among mechanically ventilated patients. However, this finding is based on studies with small sample sizes and requires further validation through larger, multicenter investigations. In clinical practice, the potential risks of this drug interaction should be carefully evaluated.

## 1 Introduction

Mechanical ventilation is a key supportive measure for treating patients with acute respiratory failure in the intensive care unit (ICU), and effective pain relief and anti-infective treatment are important links to ensure the success of mechanical ventilation. Fentanyl, as a potent μ-opioid receptor agonist, is a commonly used analgesic in the ICU [1,2]and is also widely used for analgesia and sedation in mechanically ventilated patients [3,4]. Linezolid has been approved for the treatment of multiple infections, including infections caused by vancomycin-resistant Enterococcus faecalis; hospital-acquired pneumonia caused by Staphylococcus aureus; complex skin and skin structure infections (cSSSIs); uncomplicated cSSSIs caused by methicillin-sensitive Staphylococcus aureus or Streptococcus pyogenes; and community-acquired pneumonia caused by Streptococcus pneumoniae. Case reports indicate linezolid exhibits susceptibility against bacilli. For ICU patients, methicillin-resistant Staphylococcus aureus (MRSA) and vancomycin-resistant Enterococcus (VRE) represent the two most prevalent Gram-positive pathogens. Consequently, linezolid is frequently employed to treat infected ICU patients [5–7]. Linezolid, as a weak monoamine oxidase inhibitor (MAOI), can increase the concentration of serotonin (5-HT) in the central nervous system by inhibiting its metabolism [8–10]. Fentanyl and its analogues have been shown to directly inhibit 5-HT transporters and norepinephrine transporters, thereby enhancing 5-HTergic neurotransmission [11–13]. This synergistic effect of pharmacodynamics may induce serotonin syndrome, characterized by autonomic nervous system dysfunction, neuromuscular abnormalities, and changes in mental state [14,15]. Although SS has a low recognition rate in clinical practice, its severe manifestations, such as hypertensive crisis, pulmonary edema, and multiple organ failure, may significantly increase the risk of mortality in patients [16–18].

At present, there is limited research on the impact of fentanyl combined with linezolid on the prognosis of mechanically ventilated patients, especially in terms of in-hospital mortality. Although case reports and systematic reviews suggest that the combination of the two may increase the risk of SS [19–21], large-scale clinical data is still lacking. Therefore, based on the MIMIC-IV database, this study aims to explore the impact of fentanyl combined with linezolid on in-hospital mortality in mechanically ventilated patients and analyze potential risk factors to provide a basis for rational drug use in clinical practice.

## 2 Methods

### 2.1 Data source

We conducted a retrospective cohort study using publicly available clinical data MIMIC-IV [22]. MIMIC-IV version 2.2 is an electronic health record database that includes > 50,000 patients admitted to ICU at the Beth Israel Deaconess Medical Center (Boston, MA, USA) from 2008 to 2019. The Institutional Review Board at the Beth Israel Deaconess Medical Center granted a waiver of informed consent and approved the sharing of the research resource, and Author Xiao-Qing Yi passed the online training courses and exams (certification number: 59888607).

### 2.2 Study population

There were 63,916 patients receiving mechanical ventilation in the MIMIC-IV 2.2 database. We excluded those who did not use fentanyl, had used other opioid drugs, had used antidepressants, and had been in the ICU for less than 24 hours. If a patient is admitted to the ICU multiple times, only the first patient admitted to the ICU will be analyzed.

### 2.3 Exposure and outcomes

The population we studied was patients who used fentanyl during mechanical ventilation, divided into three groups: patients who were not exposed to linezolid, patients who were simultaneously exposed to linezolid, and patients who were exposed to linezolid within 14 days. The use of fentanyl and linezolid was extracted from the “inputevents” table in the ICU. The outcome is a hospital all-cause mortality rate.

### 2.4 Data extraction

Extract data from the MIMIC IV database using structured query language. Collected data included age, sex, ethnicity, and BMI. We extracted laboratory test results 24 hours prior to ICU admission, including WBC, lymphocytes, eosinophils, neutrophils, ALT, AST, creatinine, and glucose. We extracted the patient’s comorbidities, including chronic pulmonary disease, rheumatic disease, liver disease, peptic ulcer disease, malignant cancer, myocardial infarct, peripheral vascular disease, cerebrovascular disease, diabetes, and renal disease. Extract infection-related indicators, including suspected infection, positive culture, and use of antibiotics. We also extracted GCS, body temperature, and SIRS.

### 2.5 Statistical analysis

The study cohort was divided into three groups: the group receiving linezolid simultaneously (Simultaneously Lzd), the group receiving linezolid within 14 days (Within 14 Days Lzd), and the group that did not use linezolid (NO-Lzd). The values of BMI greater than 100 or less than 10 were regarded as abnormal values, and the variables with missing values >50% were not included in the study. Multiple imputation was used to estimate the missing value of each variable [23,24]. The mean (standard deviation [SD]) and median (interquartile range [IQR]) should be used to describe normal and non-normal distribution data, respectively [25]. Cox proportional hazards models were created to generate the hazard ratio (HR) with 95% conﬁdence interval (CI) for the in-hospital all-cause mortality. For all analyses, a two-tailed P < 0.05 was considered statistically signiﬁcant. Finally, according to age, gender, race, BMI, liver disease, and kidney disease were analyzed by the Cox regression model. In addition, likelihood ratio tests were performed to explore potential interactions between subgroups.

All statistical analyses were performed using Free Statistics software version 1.9 and the R software packages (http://www.R-project.org, The R Foundation).

## 3 Results

### 3.1 Patient selection

**Fig 1:**
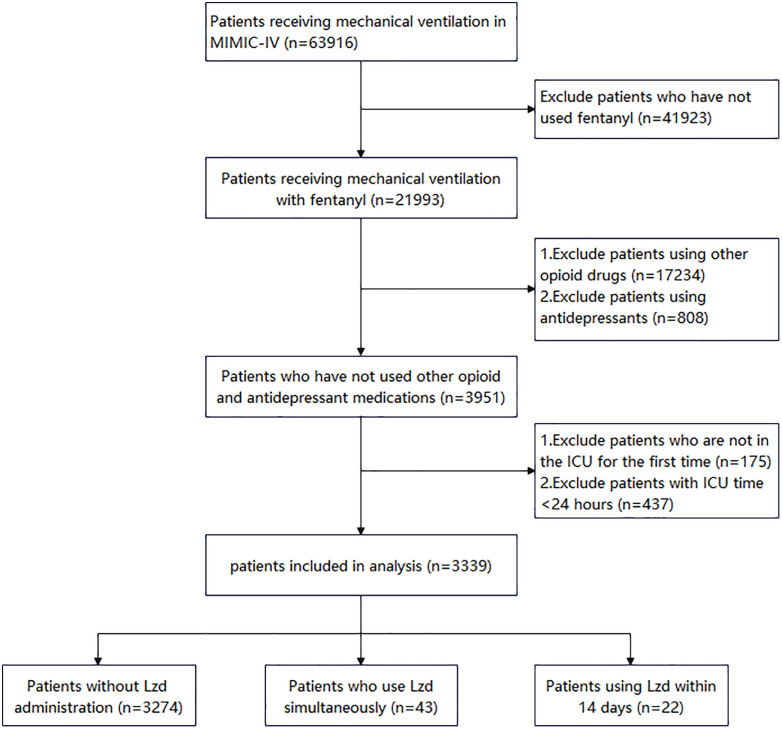
Flow chart of patient selection. MIMIC IV, Multiparameter Intelligent Monitoring in Intensive Care Database IV; ICU, intensive care unit.

We retrieved 63,916 patients receiving mechanical ventilation and 21,993 patients receiving fentanyl from the MIMIC-IV database. After excluding patients who were not included in the study, the final study population consisted of 3,339 patients, including 3,274 who did not use linezolid, 43 who also used linezolid, and 22 who used linezolid within 14 days (Fig 1).

### 3.2 Cohort characteristics

Table 1 shows the baseline characteristics of the Simultaneously Lzd group, Within 14 Days Lzd group, and NO Lzd group. Overall, the bacterial culture rates of patients in the Simultaniously Lzd group 7 (16.7%) and Within 14 Days Lzd group 9 (40.9%) were higher than those in the NO Lzd group 307 (12.2%) [p < 0.001]. The Simultaneously Lzd group 24 (55.8%) and the Within 14 Days Lzd group 8 (36.4%) had more patients with SIRS = 4 than the NO Lzd group 811 (24.8%) [p < 0.001]. In laboratory indicators, the Simultaneously Lzd group (1.9) and Within 14 Days Lzd group (1.3) had higher creatinine levels than the NO Lzd group (1.1) [p < 0.001]. There was a statistical difference among the three groups in the three complications of liver disease, kidney disease, and diabetes [p < 0.05].

**Table 1:** Baseline characteristics of entire cohort.

Variables	Overall (n = 3339)	NO-Lzd (n = 3274)	Simultaneously Lzd (n = 43)	Within 14 Days Lzd (n = 22)	P value
**Demographics**					
Age, year	64.6 ± 17.3	64.6 ± 17.3	64.1 ± 13.8	66.8 ± 17.0	0.825
Gender, Male, n (%)	2054 (61.5)	2012 (61.5)	27 (62.8)	15 (68.2)	0.799
Race white, n (%)	1825 (54.7)	1791 (54.7)	23 (53.5)	11 (50)	0.896
BMI, kg/m2	29.1 ± 8.3	29.1 ± 8.3	32.9 ± 12.1	28.7 ± 8.3	0.011
**State of illness**					
Temperature	36.9 ± 0.7	36.9 ± 0.7	36.9 ± 0.9	37.0 ± 0.8	0.674
First-day gcs	11.1 ± 4.2	11.1 ± 4.2	9.2 ± 4.8	9.1 ± 3.8	0.002
SIRS, n (%)					0.001
0	26 (0.8)	26 (0.8)	0 (0)	0 (0)	
1	272 (8.1)	271 (8.3)	1 (2.3)	0 (0)	
2	857 (25.7)	851 (26)	4 (9.3)	2 (9.1)	
3	1341 (40.2)	1315 (40.2)	14 (32.6)	12 (54.5)	
4	843 (25.2)	811 (24.8)	24 (55.8)	8 (36.4)	
Suspected infection, n (%)					1
NO	79 (3.0)	78 (3)	1 (2.3)	0 (0)	
YES	2578 (97.0)	2514 (97)	42 (97.7)	22 (100)	
Positive culture, n (%)					0.002
NO	2255 (87.5)	2207 (87.8)	35 (83.3)	13 (59.1)	
YES	323 (12.5)	307 (12.2)	7 (16.7)	9 (40.9)	
Antibiotic, n (%)					< 0.001
NO	682 (20.4)	682 (20.8)	0 (0)	0 (0)	
YES	2657 (79.6)	2592 (79.2)	43 (100)	22 (100)	
**Laboratory Examination**					
WBC	11.4 (8.3, 15.7)	11.3 (8.3, 15.7)	13.3 (8.1, 20.1)	13.4 (10.8, 16.7)	0.085
lymphocytes	16.1 (1.1, 109.2)	14.0 (1.1, 109.2)	8.2 (0.7, 84.6)	78.7 (1.5, 138.4)	0.139
eosinophils	0.2 (0.0, 5.3)	0.2 (0.0, 5.3)	0.1 (0.0, 4.5)	1.7 (0.0, 9.3)	0.507
neutrophils	129.6 (9.8, 926.9)	117.2 (9.8, 917.7)	54.6 (9.1, 1201.4)	833.4 (50.4, 1168.9)	0.027
ALT	30.5 (17.0, 71.0)	30.5 (17.0, 71.0)	38.0 (19.6, 107.0)	38.5 (18.4, 64.8)	0.69
AST	45.0 (26.0, 110.5)	45.0 (26.0, 109.4)	63.0 (29.5, 185.1)	41.9 (32.7, 108.2)	0.374
creatinine	1.1 (0.8, 1.9)	1.1 (0.8, 1.9)	1.9 (1.2, 4.1)	1.3 (0.8, 2.0)	< 0.001
Glucose	134.0 (109.4, 173.8)	134.0 (109.4, 173.6)	142.4 (108.8, 188.2)	129.5 (113.7, 168.8)	0.551
**Comorbidities**					
Chronic pulmonary disease, n (%)	871 (26.1)	853 (26.1)	13 (30.2)	5 (22.7)	0.773
Rheumatic disease, n (%)	85 (2.5)	83 (2.5)	1 (2.3)	1 (4.5)	0.607
Liver disease, n (%)	637 (19.1)	618 (18.9)	15 (34.9)	4 (18.2)	0.034
Peptic ulcer disease, n (%)	192 (5.8)	188 (5.7)	2 (4.7)	2 (9.1)	0.673
Malignant cancer, n (%)	319 (9.6)	313 (9.6)	4 (9.3)	2 (9.1)	1
Myocardial infarct, n (%)	702 (21.0)	688 (21)	7 (16.3)	7 (31.8)	0.338
Peripheral vascular disease, n (%)	319 (9.6)	313 (9.6)	4 (9.3)	2 (9.1)	1
Cerebrovascular disease, n (%)	509 (15.2)	501 (15.3)	3 (7)	5 (22.7)	0.193
Diabetes, n (%)	1003 (30.0)	970 (29.6)	18 (41.9)	15 (68.2)	< 0.001
Renal disease, n (%)	776 (23.2)	751 (22.9)	16 (37.2)	9 (40.9)	0.013
**Events**					
In-hospital mortality, n (%)	776 (23.2)	751 (22.9)	16 (37.2)	9 (40.9)	0.013
LOS hospital, days (IQR)	7.6 (4.1, 13.0)	7.6 (4.1, 13.0)	9.4 (3.2, 20.2)	13.5 (8.3, 19.7)	0.007

gcs, glasgow coma scale; SIRS, systemic inflammatory response syndrome; LOS, length of stay.

### 3.3 In-hospital mortality rate results

The overall in-hospital mortality rate was 23.2% (776/3339). Table 1 shows that the in-hospital mortality rate in the Simultaniously Lzd group was 37.2% (16/43), 40.9% (9/22) in the Within 14 Days Lzd group, and 22.9% (751/3274) in the NO Lzd group (Table 1). In the multivariate Cox regression analysis, we adjusted five models, including covariates that showed significant differences (P < 0.05) in the univariate analysis (Table 2). Compared with the NO Lzd group, in the unadjusted model, the Simultaniously Lzd group had an increased risk of in-hospital mortality by 119% (HR, 2.19; 95% CI, 1.43–3.35; P < 0.001) (Table 3). After adjusting for confounding factors such as age, gender, race, BMI, and covariates that showed significant differences (P < 0.05) in univariate analysis, the HR of the Simultaneous Lzd group in multivariate analysis was 1.56 (95% CI, 1–2.43; p = 0.049) (Table 3). There was no statistically significant difference in in-hospital mortality rate between the Lzd group within 14 days and the NO Lzd group (p > 0.5).

**Table 2:** Univariate Cox regression analysis of in-hospital mortality rate.

Variable	HR(95%CI)	P(Wald’s test)
age	1.0031 (0.9986,1.0076)	0.18
Male	0.94 (0.81,1.09)	0.392
Withe	0.8 (0.69,0.92)	0.002
BMI (cont. var.)	1.0028 (0.9945,1.0111)	0.51
antibiotic	2.24 (1.7,2.96)	< 0.001
firstday.gcs	0.96 (0.94,0.98)	< 0.001
temperature_mean	0.65 (0.6,0.7)	< 0.001
Suspected infection	0.9986 (0.6397,1.5587)	0.995
Positive culture	1.61 (1.31,1.96)	< 0.001
SIRS: ref. = 0		
1	1.33 (0.17,10.17)	0.783
2	3.56 (0.5,25.53)	0.206
3	5.85 (0.82,41.65)	0.078
4	10.98 (1.54,78.22)	0.017
Chronic pulmonary disease	1.06 (0.9,1.25)	0.461
Rheumatic disease	1.29 (0.86,1.92)	0.218
Liver disease	1.75 (1.49,2.05)	< 0.001
Peptic ulcer disease	0.65 (0.44,0.96)	0.032
Malignant cancer	1.54 (1.25,1.91)	< 0.001
Myocardial infarct	1.35 (1.14,1.59)	< 0.001
Peripheral vascular disease	1.55 (1.26,1.92)	< 0.001
Cerebrovascular disease0	0.81 (0.66,0.99)	0.042
Diabetes	0.94 (0.8,1.1)	0.438
Renal disease	1.17 (0.99,1.38)	0.059
WBC	1.02 (1.02,1.02)	< 0.001
Lymphocytes	1.0001 (1,1.0002)	< 0.001
Eosinophils	0.98 (0.97,0.99)	< 0.001
Neutrophils	0.9999 (0.9998,1.0001)	0.305
ALT	1.0001 (1.0001,1.0002)	< 0.001
AST	1.0001 (1.0001,1.0002)	< 0.001
Creatinine	1.12 (1.09,1.15)	< 0.001
Glucose	1 (1,1)	0.163
Simultaniously Lzd	2.19 (1.43,3.35)	< 0.001
Within 14 Days Lzd	1.27 (0.63,2.54)	0.506

**Table 3:** Multivariable Cox regression analysis for in-hospital mortality.

Categories	Model I		Model II		Model III		Model IV		Model V	
	HR (95%CI)	P-value	HR (95%CI)	P-value	HR (95%CI)	P-value	HR (95%CI)	P-value	HR (95%CI)	P-value
**In-hospital mortality**										
NO-Lzd	1(Ref)		1(Ref)		1(Ref)		1(Ref)		1(Ref)	
Simultaneously Lzd	2.19 (1.43 ~ 3.35)	<0.001	2.21 (1.44 ~ 3.39)	<0.001	1.58 (1.02 ~ 2.46)	0.04	1.59 (1.02 ~ 2.46)	0.04	1.56 (1 ~ 2.43)	0.049
Within 14 Days Lzd	1.27 (0.63 ~ 2.54)	0.506	1.27 (0.63 ~ 2.55)	0.504	0.87 (0.43 ~ 1.75)	0.698	0.97 (0.48 ~ 1.96)	0.936	1.01 (0.5 ~ 2.05)	0.968

Model I: did not adjust any variables.

Model II: adjusted for age, gender, race, BMI.

Model Ⅲ: adjusted for II covariates, temperature, firstday gcs, positive culture, Sirs, antibictic.

Model Ⅳ: adjusted for Ⅲ covariates, WBC, lymphocytes, eosinophils, ALT, AST, creatinine.

Model Ⅴ: adjusted for Ⅳ covariates, liver disease, peptic ulcer disease, malignant cancer, myocardial infarct, peripheral vascular disease, cerebrovascular disease.

### 3.4 Subgroup analyses

**Fig 2:**
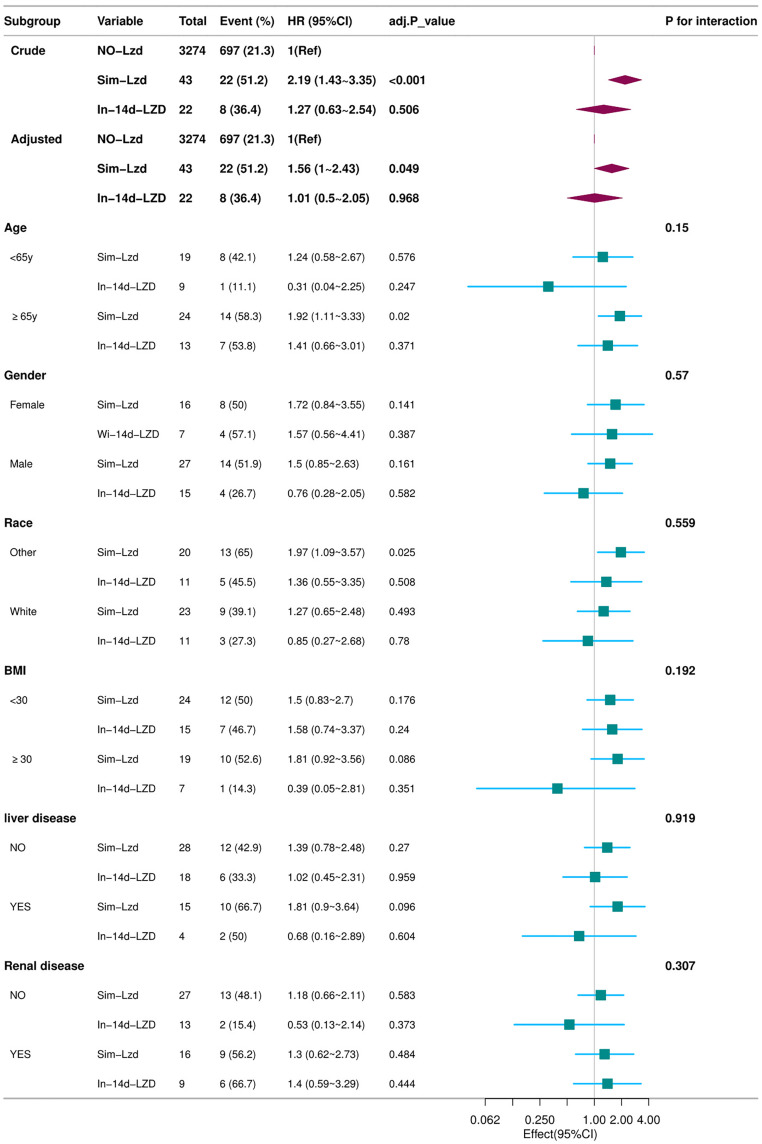
Subgroup analyses for in-hospital mortality. The multivariable Cox proportional hazards model was adjusted for temperature, firstday gcs, positive culture, sirs, antibictic, wbc, lymphocytes, eosinophils, alt, ast, creatinine, peptic ulcer disease, malignant cancer, myocardial infarct, peripheral vascular disease, cerebrovascular disease.

Fig 2 shows the subgroup analysis results of in-hospital mortality rate. All subgroup analyses indicate that regardless of baseline patient characteristics, the Simultaneiously Lzd group increases in-hospital mortality (HR > 1). Especially in the subgroup analysis of age ≥ 65, there was a statistically significant increase in the risk of in-hospital mortality (HR, 1.92; 95% CI, 1.11–3.33; P = 0.02).

## 4 Discussion

The study findings indicate that concurrent use of fentanyl and linezolid may be associated with increased in-hospital mortality among mechanically ventilated patients. However, this association (adjusted hazard ratio 1.56) should be interpreted with caution, as it is marginally statistically significant (95% CI: 1.00–2.43, P = 0.049) and based on limited analytical data. Most critically, the small number of patients (n = 43) and outcome events (16 deaths) in the combination group rendered our multivariate model statistically unstable and underpowered. Furthermore, patients receiving this combination had more severe baseline conditions, higher creatinine levels, and higher SIRS scores, along with a greater prevalence of comorbidities such as hepatic and renal disease. Despite statistical adjustments, substantial residual confounding due to indications may persist, suggesting that the observed mortality signal may reflect underlying disease severity as well as potential drug interactions. Therefore, we interpret this finding as a hypothesis-generating and preliminary result.

The pharmacological characteristics of these two drugs provide a plausible yet unproven biological rationale for explaining potential risks: fentanyl reduces serotonin reuptake by inhibiting the serotonin transporter, while linezolid, as a weak MAOI, decreases serotonin metabolism [10,13]. This dual mechanism may lead to excessive serotonin levels, increasing the risk of SS or related complications such as autonomic instability, hyperthermia, or multiple organ failure [9,18]. Furthermore, fentanyl respiratory depressant effects may be exacerbated by linezolid neurotoxicity, particularly in mechanically ventilated patients experiencing weaning difficulties or ventilatory failure [21]. However, it must be emphasized that our study lacks direct measurements of serotonin levels and clinical diagnoses of SS, rendering the proposed mechanism speculative. Based solely on our data, the observed mortality association cannot be fully attributed to this interaction.

Subgroup analysis revealed that patients aged 65 years or older were particularly vulnerable, exhibiting a statistically significant increase in mortality risk (HR = 1.92, 95% CI = 1.11–3.33, P = 0.02). This aligns with case reports describing mortality outcomes in elderly patients [26,27]. However, this subgroup analysis is also severely limited by its small sample size and should be regarded as an exploratory finding.

In conclusion, this study identifies a preliminary signal that warrants further investigation but fails to provide robust evidence for a causal link between simultaneous fentanyl-linezolid use and increased mortality. The primary contribution of this work is to highlight a complex clinical scenario where therapeutic necessity and potential risk must be balanced and to underscore the critical need for more definitive research. Future investigations should ideally be large, multi-center prospective studies or well-designed target trial emulations that are adequately powered to account for the significant baseline confounding and the low prevalence of this drug combination. Until such evidence is available, clinicians should remain aware of the potential risks discussed in the pharmacological literature and maintain a high level of vigilance when the concurrent use of these medications is unavoidable, particularly in older and more vulnerable patients.

## 5 Limitations

This study has several limitations. First, its retrospective design precludes causal inferences, and unmeasured confounders (e.g., severity of infection or undiagnosed SS) may have influenced outcomes. Second, the small sample size of the linezolid-exposed groups (n = 43 and n = 22) limits statistical power, particularly for subgroup analyses. Third, the MIMIC-IV database lacks detailed pharmacodynamics data (e.g., serotonin levels or SS diagnoses), which would strengthen mechanistic conclusions. Future prospective studies should incorporate these metrics to validate the observed association.

## 6 Conclusion

Studies indicate that concurrent use of fentanyl and linezolid in mechanically ventilated patients is associated with increased in-hospital mortality. However, this finding is based on studies with small sample sizes and requires validation through larger, multicenter investigations. This association may relate to drug interactions, necessitating further research to elucidate the underlying mechanisms and explore risk mitigation strategies. In clinical practice, the potential risks of this drug interaction should be assessed, and alternative medications should be prioritized.

## Supporting information

S1 Data Data - 12.17. (XLSX)

## References

[1] Martin J, Parsch A, Franck M, Wernecke KD, Fischer M, Spies C. Practice of sedation and analgesia in German intensive care units: results of a national survey. Crit Care. 2005;9(2):R117-23. doi: 10.1186/cc3035 15774043 PMC1175921

[2] Soliman HM, Mélot C, Vincent JL. Sedative and analgesic practice in the intensive care unit: the results of a European survey. Br J Anaesth. 2001;87(2):186–92. doi: 10.1093/bja/87.2.186 11493487

[3] Casamento A, Bellomo R. Fentanyl versus morphine for analgo-sedation in mechanically ventilated adult ICU patients. Crit Care Resusc. 2019;21(2):76–83. doi: 10.1016/s1441-2772(23)00666-x 31142236

[4] Aoki Y, Kato H, Fujimura N, Suzuki Y, Sakuraya M, Doi M. Effects of fentanyl administration in mechanically ventilated patients in the intensive care unit: a systematic review and meta-analysis. BMC Anesthesiol. 2022;22(1):323. doi: 10.1186/s12871-022-01871-7 36271330 PMC9585711

[5] Yue J, Dong BR, Yang M, Chen X, Wu T, Liu GJ. Linezolid versus vancomycin for skin and soft tissue infections. Cochrane Database Syst Rev. 2016;2016(1):CD008056. doi: 10.1002/14651858.CD008056.pub3 26758498 PMC10435313

[6] Hashemian SMR, Farhadi T, Ganjparvar M. Linezolid: a review of its properties, function, and use in critical care. Drug Des Devel Ther. 2018;12:1759–67. doi: 10.2147/DDDT.S164515 29950810 PMC6014438

[7] Liu BM, Beck EM, Fisher MA. The Brief Case: Ventilator-Associated Corynebacterium accolens Pneumonia in a Patient with Respiratory Failure Due to COVID-19. J Clin Microbiol. 2021;59(9):e0013721. doi: 10.1128/JCM.00137-21 34406882 PMC8372998

[8] Gupta V, Karnik ND, Deshpande R, Patil MA. Linezolid-induced serotonin syndrome. BMJ Case Rep. 2013;2013:bcr2012008199. doi: 10.1136/bcr-2012-008199 23513014 PMC3618736

[9] Elbarbry F, Moshirian N. Linezolid-associated serotonin toxicity: a systematic review. Eur J Clin Pharmacol. 2023;79(7):875–83. doi: 10.1007/s00228-023-03500-9 37129603

[10] Wali HA. Linezolid and serotonin syndrome. J Int Med Res. 2025;53(2):3000605251315355. doi: 10.1177/03000605251315355 39932284 PMC12059600

[11] Wittmann M, Schaaf T, Peters I, Wirz S, Urban BW, Barann M. The effects of fentanyl-like opioids and hydromorphone on human 5-HT3A receptors. Anesth Analg. 2008;107(1):107–12. doi: 10.1213/ane.0b013e31817342c2 18635474

[12] Barann M, Stamer UM, Lyutenska M, Stüber F, Bönisch H, Urban B. Effects of opioids on human serotonin transporters. Naunyn Schmiedebergs Arch Pharmacol. 2015;388(1):43–9. doi: 10.1007/s00210-014-1056-3 25332055

[13] Rickli A, Liakoni E, Hoener MC, Liechti ME. Opioid-induced inhibition of the human 5-HT and noradrenaline transporters in vitro: link to clinical reports of serotonin syndrome. Br J Pharmacol. 2018;175(3):532–43. doi: 10.1111/bph.14105 29210063 PMC5773950

[14] Gillman PK. Monoamine oxidase inhibitors, opioid analgesics and serotonin toxicity. Br J Anaesth. 2005;95(4):434–41. doi: 10.1093/bja/aei210 16051647

[15] Baldo BA, Rose MA. The anaesthetist, opioid analgesic drugs, and serotonin toxicity: a mechanistic and clinical review. Br J Anaesth. 2020;124(1):44–62. doi: 10.1016/j.bja.2019.08.010 31653394

[16] Shahbazi F, Shojaei L. Hypertensive Crisis Associated with Serotonin Syndrome Following Linezolid Administration: Report of Two Cases. Curr Drug Saf. 2025;20(2):241–6. doi: 10.2174/0115748863299100240507052341 38847376

[17] Shah ND, Jain AB. Serotonin syndrome presenting as pulmonary edema. Indian J Pharmacol. 2016;48(1):93–5. doi: 10.4103/0253-7613.174575 26997733 PMC4778218

[18] McGreevy JL, Piraino ST, Sandhu G. Fentanyl-Associated Serotonin Syndrome and Chest Wall Rigidity in an Intensive Care Unit Patient. Am J Ther. 2019;26(5):e612–4. doi: 10.1097/MJT.0000000000000813 29965805

[19] Samartzis L, Savvari P, Kontogiannis S, Dimopoulos S. Linezolid is associated with serotonin syndrome in a patient receiving amitriptyline, and fentanyl: a case report and review of the literature. Case Rep Psychiatry. 2013;2013:617251. doi: 10.1155/2013/617251 23533900 PMC3603624

[20] Corsini Campioli C, Barth D, Esquer Garrigos Z, Abu Saleh O, Sohail RM, Sia IG. Linezolid and fentanyl: An underrecognized drug-to-drug interaction. J Clin Pharm Ther. 2020;45(4):825–7. doi: 10.1111/jcpt.13143 32304579

[21] Mitwally H, Saad MO, Alkhiyami D, Fahmi AM, Mahmoud S, Hmoud EA, et al. Risk of serotonin syndrome in acutely ill patients receiving linezolid and opioids concomitantly: a retrospective cohort study. IJID Reg. 2022;5:137–40. doi: 10.1016/j.ijregi.2022.09.008 36324824 PMC9618969

[22] Johnson AEW, Bulgarelli L, Shen L, Gayles A, Shammout A, Horng S, et al. MIMIC-IV, a freely accessible electronic health record dataset | Scientific Data. [cited 11 Apr 2024]. Available from: https://www.nature.com/articles/s41597-022-01899-x 10.1038/s41597-022-01899-x PMC9810617 36596836

[23] Sterne JAC, White IR, Carlin JB, Spratt M, Royston P, Kenward MG, et al. Multiple imputation for missing data in epidemiological and clinical research: potential and pitfalls. BMJ. 2009;338:b2393. doi: 10.1136/bmj.b2393 19564179 PMC2714692

[24] Mackinnon A. The use and reporting of multiple imputation in medical research - a review. J Intern Med. 2010;268(6):586–93. doi: 10.1111/j.1365-2796.2010.02274.x 20831627

[25] Habibzadeh F. Statistical Data Editing in Scientific Articles. J Korean Med Sci. 2017;32(7):1072. doi: 10.3346/jkms.2017.32.7.1072 28581261 PMC5461308

[26] Sutton J, Stroup J, Som M. Linezolid-induced serotonin toxicity in a patient not taking monoamine oxidase inhibitors or serotonin receptor antagonists. Proc (Bayl Univ Med Cent). 2016;29(2):214–5. doi: 10.1080/08998280.2016.11929423 27034576 PMC4790578

[27] Bernard L, Stern R, Lew D, Hoffmeyer P. Serotonin Syndrome after Concomitant Treatment with Linezolid and Citalopram. Clin Infect Dis. 2003;36(9):1197–1197. doi: 10.1086/374558 12715317

