# Correction: The effect of fentanyl combined with linezolid on in-hospital mortality in mechanically ventilated patients: A retrospective cohort study

**DOI:** 10.1371/journal.pone.0344603

**Published:** 2026-03-09

**Authors:** Jia-Hui Niu, Xiao-Jiao Cui, Min Chen, Jin-Qi Li, Xiao-Qing Yi

There are errors in the author affiliations. The correct affiliations are as follows:

Jia-Hui Niu^2^, Xiao-Jiao Cui^1^, Min Chen^1^, Jin-Qi Li^1^, Xiao-Qing Yi^1^

**1** Department of Pharmacy, Personalized Drug Research and Therapy Key Laboratory of Sichuan Province, Sichuan Provincial People’s Hospital, University of Electronic Science and Technology of China, **2** Sichuan Academy of Medical Sciences, Sichuan Provincial People’s Hospital, School of Medicine,University of Electronic Science and Technology of China, Chengdu, China, No. 32, West Second Section, First Ring Road, Chengdu, China.
